# The dermatologists’ case for the bidet

**DOI:** 10.1097/JW9.0000000000000103

**Published:** 2023-08-10

**Authors:** Yoseph Dalia, Hebah Al Khateeb, Tejesh Patel

**Affiliations:** a Department of Dermatology, University of Tennessee Health Science Center, Memphis, Tennessee; b Department of Dermatology, University of Tennessee Health Science Center, College of Medicine, Memphis, Tennessee

**Keywords:** bidet, caregiver, climate change, hidradenitis, hygiene, perianal disease, public health

**What is known about this subject in regard to women and their families?**

- There are several dermatological conditions that disproportionally affect the genital and perianal areas of women.
- Dry toilet paper and wet wipes can potentially aggravate these conditions.
- Toileting assistance is a major role of primary caregivers, and a majority of primary caregivers are women.
- Climate change is a health threat to women and their families.

**What is new from this article as messages for women and their families?**

- Bidets are commonly used outside the Western world.
- Bidets can be recommended as an alternative to toilet paper or wet wipes in patients with perianal dermatological conditions.
- Bidets can increase toileting independence in those with disabilities.
- Bidets are more environmentally friendly than toilet paper.
- There are affordable, easy-to-install bidet options.

## Dear Editors,

### Introduction

In this editorial, we contend that dermatologists should understand bidets and feel comfortable recommending their use. Primarily, they should be aware of the medical benefits they offer. Additionally, physicians should consider the environmental benefits as stewards of the environment. Finally, being aware of the commonality of bidet use outside of American culture can be considered part of a physician’s duty to cultural humility.

### Medical benefits

Several dermatologic conditions affect the perianal region, including but not limited to eczema, hidradenitis suppurativa, psoriasis, contact dermatitis, and lichen sclerosus. Due to the sensitive skin in the genital area, symptoms of diseases such as pruritus and burning can be more pronounced with toilet paper or irritant- or allergen-containing wet wipes. Using water to gently cleanse the area could alleviate the shortcomings of dry toilet paper and wet wipes. Bidets can also be useful for patients with a body habitus or disability that prevents them from independently cleaning after toileting. In a study performed with patients in a stroke rehabilitation unit, subjects who used an electric bidet had an increased quality of life by improving toileting independence and cleanliness after defecation.^1^ Physicians can recommend the use of bidets as part of the symptomatic management of various diseases within the perianal region.

### Environmental benefits

Climate change, biodiversity loss, and worsening natural disasters represent a threat to both the environmental and human health. Regardless of age, ethnicity, socioeconomic status, or geographical location, its negative health impacts are deleterious. Vulnerable populations such as children, elderly, minorities, and low socioeconomic status populations are disproportionately affected. The growing link between climate change and its impact on patients has led the medical community, including dermatologists, to address our role in the crisis. Several articles have been published in *Journal of the American Medical Association Dermatology* as well as the *International Journal of Women’s Dermatology*, emphasizing that major changes in climate have specifically affected dermatological diseases.^2–4^ For these reasons, it is essential that medical professionals remain wellinformed about the climate crisis and invest in resources to prevent the exacerbation of health risks for our patients’ wellbeing. A sustainable resource that has the potential to have significant environmental benefits is the use of bidets. Toilet paper requires the use of 15 million trees to produce the 36 billion rolls of toilet paper Americans use annually. On average, it takes 36 gallons of water to produce a single roll of toilet paper. A bidet uses an estimated 1/8th of a gallon per use.^5^ Moreover, bidets can be used indefinitely, thus reducing carbon emissions related to transport and packaging that toilet paper requires. Physicians can be assured that they are making an environmentally conscious recommendation.

### Conclusion

In conclusion, bidets offer an alternative to toilet paper from a health, hygiene, and environmental standpoint. Bidet use is the predominant form of perianal hygiene in the Eastern world. Therefore, we hope that this editorial will serve to educate North American dermatologists on the benefits a bidet can provide to patients. Table 1 provides a brief description of the various types of bidets.

**Table 1:** Information of common bidet styles

	Description	Difficulty of installation	Requires increased bathroom space	Limitations of use
Standalone	A separate unit from the toilet, containing its own bowl and faucet.	High	Yes	Requires patients to physically transfer from toilet seat to bidet.
Handheld bidet/hose	A separate handheld hose with a shower head like nozzle.	Medium	Maybe	Patients who already strain to reach perianal area may have issues with directing nozzle to that region.
Electric bidet attachment	A bidet that attaches directly under the existing toilet seat.	Low	No	Fixed positioning on the toilet may limit the area to be washed.

## Conflicts of interest

Y. D.: Received 20 sample products intended for patient use from the bidet company, “TUSHY.” There are no conflicts of interest for the remaining authors.

## Funding

None.

## Study approval

N/A
